# High efficiency DBR assisted grating chirp generators for silicon nitride fiber-chip coupling

**DOI:** 10.1038/s41598-019-55140-8

**Published:** 2019-12-11

**Authors:** Siddharth Nambiar, Praveen Ranganath, Rakshitha Kallega, Shankar Kumar Selvaraja

**Affiliations:** Center for Nanoscience and Engineering, Indian Institute of Science, Bengaluru, 560012 India

**Keywords:** Silicon photonics, Photonic devices, Integrated optics

## Abstract

Silicon Nitride (*SiN*) is emerging as a promising material for a variety of integrated photonic applications. Given its low index contrast however, a key challenge remains to design efficient couplers for the numerous platforms in *SiN* photonics portfolio. Using a combination of bottom reflector and a chirp generating algorithm, we propose and demonstrate high efficiency, grating couplers on two distinct *SiN* platforms. For a partially etched grating on 500 nm thick *SiN*, a calculated peak efficiency of −0.5 dB/coupler is predicted, while for a fully etched grating on 400 nm thick *SiN*, an efficiency of −0.4 dB/coupler is predicted. Experimentally measured coupling efficiencies are observed to be −1.17 and −1.24 dB/coupler for the partial and fully etched grating couplers respectively in the C-L band region. Furthermore, through numerical simulations, it is shown that the chirping algorithm can be implemented in eight additional combinations comprising *SiN* film thickness between 300–700 nm as well as alternate claddings, to achieve a per coupler loss between −0.33 to −0.65 dB.

## Introduction

Silicon photonics is a key technology enabler for building complex optical components for a host of applications such as high performance computing, communications as well as on-chip sensing. Moreover, the mature complementary metal-oxide semiconductor (CMOS) foundry processes provides further impetus for a scalable, low cost and high volume means to integrate electronics and photonics functionalities on a single chip^1–3^. From the materials perspective, there are primarily two CMOS compatible photonic platforms which are Silicon-on-insulator (SOI) and Silicon Nitride (*SiN*). The former is a widely researched platform on which a variety of active and passive devices have been implemented till date. On the other hand, a lot of interest has been brewing in the field of *SiN* photonics, especially as an alternative integrated platform^4–7^. Several reasons are attributed to this, such as *SiN* exhibiting a wideband spectral transparency encompassing visible to mid-infrared, having higher tolerance to fabrication imperfections owing to a moderate index contrast and its near total absence of two photon absorption. Such vast potential has paved way for the emergence of several *SiN* platforms that cater to applications ranging from passive wavelength division multiplexing (WDM), spectroscopy, sensing as well as integrated nonlinear photonics. The choice of *SiN* thickness depends largely on its targeted spectrum as well as application. Lower waveguide film thickness is generally preferred for passive WDM as well as sensing applications^8–11^ while thicker films are used for nonlinear photonic applications^12–15^.

Fiber-chip coupling is a critical aspect of any photonic integrated circuit. There are principally two ways of enabling this task, which are in-plane and out-of-plane coupling. The former typically consists of edge couplers and is known to deliver high coupling efficiency as well as a broad bandwidth. However it also requires flat cleaved, polished facets and precise alignment. The latter consists of surface diffractive features imprinted on the waveguiding layer, that is used to phase match the incoming fiber mode with that of the propagating waveguide mode.

Despite offering a comparatively lower coupling and bandwidth, surface gratings are generally preferred over edge-couplers to facilitate fiber to waveguide coupling, owing to ease of fabrication and relaxed alignment tolerances. High efficiency grating couplers on SOI have been reported using a variety of design strategies^16–22^. Achieving a similar feat on *SiN* is cumbersome given its moderate index-contrast. Nevertheless, several key demonstrations have been recently reported on improving efficiency of *SiN* gratings. Broadly speaking, the grating efficiency depends on two factors, first of which is directionality, that quantifies total power scattered upwards and the second is field overlap with the fiber mode. A generic grating suffers from poor directionality due to a substantial portion of power being leaked to the substrate. One way to improve upon the directionality is to implement a double-etched grating structure. A two level highly directional, staircase grating was reported in^23^ with an efficiency of −1.5 dB, on a standalone 600 nm thick *SiN* film. Similarly, a bi-layer, double-etched grating coupler was demonstrated in^24^ with an efficiency of −2.56 dB on 220 nm thick *SiN*. A second way to improve directionality is by hybridizing *SiN* with a high index platform like SOI. High bandwidth gratings have been reported on such *SiN*-SOI platforms, such as a composite grating of *Si* and *SiN*, demonstrated in^25^ on 400 nm thick *SiN* with a coupling of −1.3 dB and a 1 dB bandwidth of 80 nm. Likewise a deep-etched *SiN*-SOI grating was reported in^26^ with a coupling of −2.5 dB and 67 nm 1 dB bandwidth. A third way to enhance directionality is by incorporating a highly reflective bottom mirror like a Bragg stack^27–29^ or a metal layer^20,21^. When a bottom mirror is placed at an optimal separation from the slab, constructive interference occurs between up-scattered and downward reflected light which suppresses substrate leakage. In^29^, we reported enhanced efficiency of uniform *SiN* grating couplers, incorporated with a bottom Bragg mirror. In this work, we shall extend the scheme to non-uniform gratings. By using a combination of a bottom reflector and a chirping generator, we show a sub-dB coupling loss on two distinct platforms, which are, a 500 nm thick *SiN* with an air cladding and a 400 nm thick *SiN* on *SiO*_2_ cladding.

The total fiber to waveguide coupled output power *P*_*CE*_, can be determined through the following expression,

1 $${P}_{{}_{CE}}=\eta {P}_{diff}$$

where *P*_*diff*_ is the proportion of upper diffracted power (or commonly referred as directionality). $\eta$, is the overlap integral between diffracted grating and fiber mode fields. $\eta$ can be further evaluated from^20,30^ as,

2 $$\eta =\frac{|\int {\int }_{-\infty }^{\infty }\,{E}_{gr}{E}_{fib}^{\ast }dA{|}^{2}}{\int {\int }_{-\infty }^{\infty }\,|{E}_{gr}{|}^{2}dA\,\int {\int }_{-\infty }^{\infty }\,|{E}_{fib}{|}^{2}dA}$$

where *E*_*fib*_ is the electric field distribution of the fiber mode and *E*_*gr*_, that of the scattered grating field. The diffracted power profile of a uniform grating is exponentially decaying along the propagation direction, can be expressed as $P(x)={P}_{0}{e}^{-2\alpha x}$, where *α* is the grating leakage parameter and *x* is mode propagation direction. Consequently, $\eta$ between a exponentially varying field distribution and a Gaussian mode is limited to around 80–85%. In order to maximize $\eta$, the leakage parameter must by engineered along the grating length so as to gradually scatter a Gaussian like profile. This variable leakage parameter *α*(*x*) can be expressed in the following form^31^ as,

3 $$\alpha (x)=\frac{{G}^{2}(x)}{2[1-{\int }_{0}^{x}\,{G}^{2}(t)dt]}$$

*G*(*x*), being the Gaussian profile scattered by the grating.

## Design and Simulation

**Figure 1:**
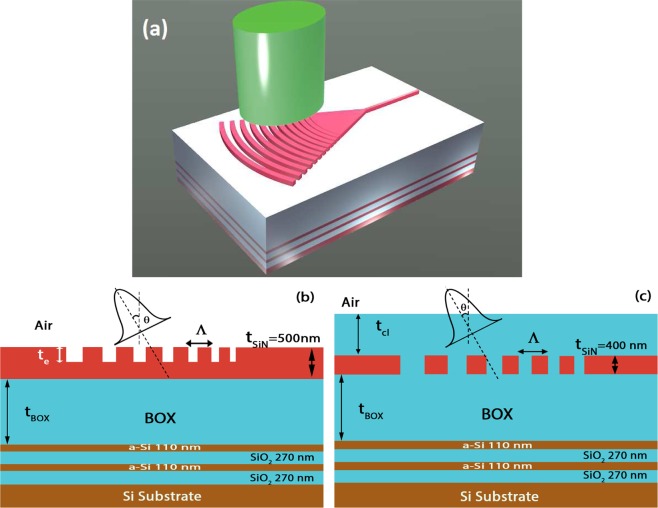
(**a**) Shows a 3D illustration of a chirped fiber-waveguide focusing grating coupler with a bottom DBR mirror. (**b**) Shows 2D schematic of grating couplers for design A and (**c**) for design B, considered in this work. As indicated, no constraints on etch depth *t*_*e*_ is imposed for design A, where as only full etched gratings are considered for design B.

Figure 1 illustrates a schematic of proposed grating structures of the two design combinations considered. The critical parameters of relevance here are $\Lambda$, which is the grating period, *f*, the fill-factor, *t*_*e*_, the etch depth, and *θ*, the incidence angle of illumination. Also, *t*_*cl*_ is the upper cladding thickness for design B, *t*_*BOX*_ is the buried oxide thickness and *t*_*SiN*_ is the waveguide core thickness. Out of these *t*_*e*_ is considered to be a variable quantity for design A and is fixed as *t*_*SiN*_ for design B where all gratings are considered fully etched. *t*_*cl*_ on the other hand, is considered to be a variable for design B. We have considered two platforms i.e. a *t*_*SiN*_ of 500 nm for design A which is interesting for nonlinear optical applications^15^ and a *t*_*SiN*_ of 400 nm for design B, which can be used for building passive photonic interconnects^8^. Beneath the BOX layer lies a distributed Bragg reflector (DBR) stack. The DBR stack, is composed of two cascaded layers of amorphous Silicon and Silicon dioxide (*a*-*Si*/*SiO*_2_) of thickness *λ*/4*N* each (*λ* is the wavelength in free-space and *N* is optical material index), which translates to 110/270 nm. Numerical simulations are performed in two dimensional-finite difference time domain (FDTD). The source is considered to be a Gaussian of mode field diameter 10.4 *μm* and is embedded a few microns above, in the air region of each grating design. A power monitor is placed in the slab waveguide, a few microns away from the grating to determine the coupling efficiency (CE). The optical indices of *Si*, *a*-*Si*, *SiO*_2_ and *SiN* are obtained from ellipsometry to be 3.46, 3.53, 1.44 and 2.015. Our goal here is to determine an optimal generator function that maximizes $\eta$ and hence coupling to waveguide. This chirp generator algorithm can be expressed in terms of a grating constructor as,

4 $$m\Lambda =\mathop{\sum }\limits_{i=1,f={f}_{s}}^{i=m.f={f}_{e}}\,[{f}_{i}\Lambda +(1-{f}_{i})\Lambda ]$$

where *f*_*s*_ and *f*_*e*_ are the start and end fill-factors respectively and *m*, the total number of grating periods. The factor ${f}_{i}\Lambda$ corresponds to the etched portion of the *i*^*th*^ grating period. The unknown period $\Lambda$ is assumed to be fixed in this generator algorithm. $\Lambda$ depends on fulfilling the Bragg phase matching condition, which is,

5 $$\Lambda =\frac{\lambda }{{N}_{gr}^{eff}-{n}_{cl}\,sin\,\theta }$$

where *n*_*cl*_ is the top cladding index and ${N}_{gr}^{eff}$, the effective grating index. Since, the fill-factor is assumed to be a variable parameter throughout the grating length, the effective grating index of the *i*^*th*^ period can then be expressed as,

6 $${N}_{igr}^{eff}={f}_{i}{n}_{et}^{eff}+(1-{f}_{i}){n}_{SiN}^{eff}$$

where ${n}_{SiN}^{eff}$ and ${n}_{et}^{eff}$ are the effective modal indices of the fundamental transverse electric (TE) mode of the slab and etched portions respectively. For design B, ${n}_{et}^{eff}$ is substituted with optical index of *SiO*_*2*_. A rigorous optimization reveals the grating constructor in terms of the chirp generator algorithm (CGA) as,

7 $$m\Lambda =\mathop{\sum }\limits_{i=1,{f}^{u}}^{i=n,{f}^{u}}\,[{f}_{i}^{u}\Lambda +(1-{f}_{i}^{u})\Lambda ]+\mathop{\sum }\limits_{i=n+1,{f}_{s}^{c}}^{i=m,{f}_{e}^{c}}\,[{f}_{i}^{c}\Lambda +(1-{f}_{i}^{c})\Lambda ]$$

In effect, the grating is composed of 2 parts, i.e., a uniform section till *n* periods and a chirped section for *m*-*n* periods. Here *f*^*u*^ corresponds to the uniform fill-factor and the chirped parameters ${f}_{s}^{c}$ and ${f}_{e}^{c}$ correspond to the start and end fill-factors. A parametric sweep is now conducted to find the optimal design parameters. For design A, *n* is found to be 3 and *m*-*n* to be 16, however in design B, *n* is 1 and *m*-*n* is 18. The optimal grating periods are 0.95 *μm* for design A and 0.99 *μm* for design B. All the relevant chirp parameters are provided in Table 1. In addition, optimal *t*_*cl*_ for design B is calculated for a thickness of 2 *μm*.

**Table 1:** CGA parameters for the two *SiN* grating design combinations.

Design	*f*_*u*_	${{\boldsymbol{f}}}_{{\boldsymbol{s}}}^{{\boldsymbol{c}}}$	${{\boldsymbol{f}}}_{{\boldsymbol{e}}}^{{\boldsymbol{c}}}$	*n*	*m*	*t*_*BOX*_ (*μm*)	*t*_*e*_ (nm)	*λ*_*max*_ (nm)	Λ (*μm*)	*θ* (*deg*)	${{\boldsymbol{f}}}_{{\boldsymbol{e}}}^{{\boldsymbol{c}}}$Λ (nm)
A	0.55	0.5	0.15	3	19	1.92	280	1550	0.95	3°	142
B	0.55	0.5	0.15	1	19	1.56	400	1560	0.99	3°	148

**Figure 2:**
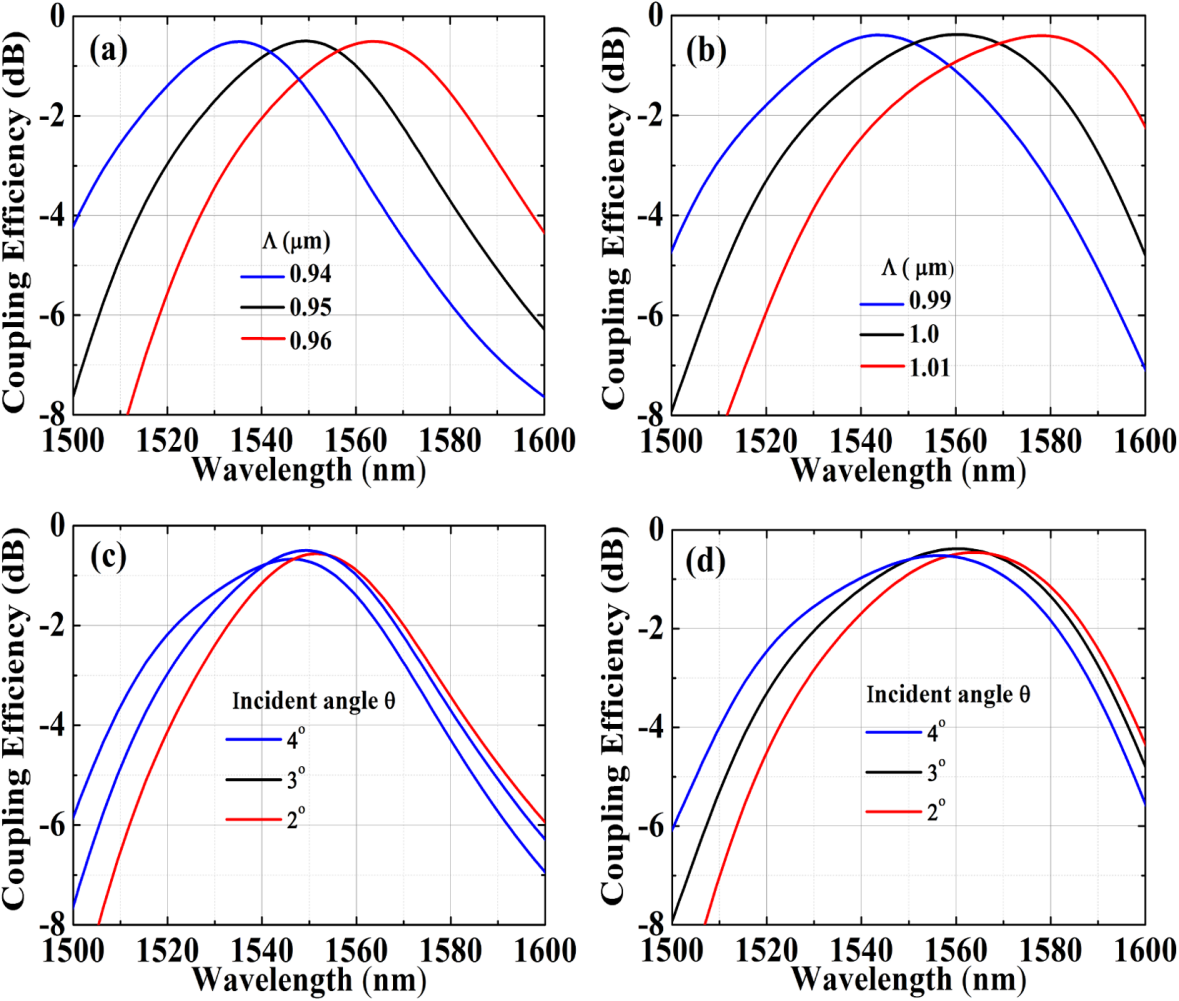
Simulated CE as a function of periods for (**a**) design A and (**b**) design B. (**c**) Shows angle dependence of CE at period Λ = 0.95 *μm* for design A. (**d**) Shows the same at Λ = 0.99 *μm* for design B.

Figure 2 shows the coupling as a function of different periods and angles for the DBR aided designs. Peak CE for design A is calculated as −0.5 dB, at incident angle 3°, at 1550 nm. For design B, peak CE is calculated as −0.38 dB at 1560 nm, also at an angle of 3°. For design A, a 10 nm increase in period results in a red shift of 14 nm for peak wavelength. For design B, this red shift is observed to be slightly higher at 16 nm. The angle sweep showcases a distinctive feature of these CGA optimized gratings, which is a high coupling observed at near vertical incidence.

**Figure 3:**
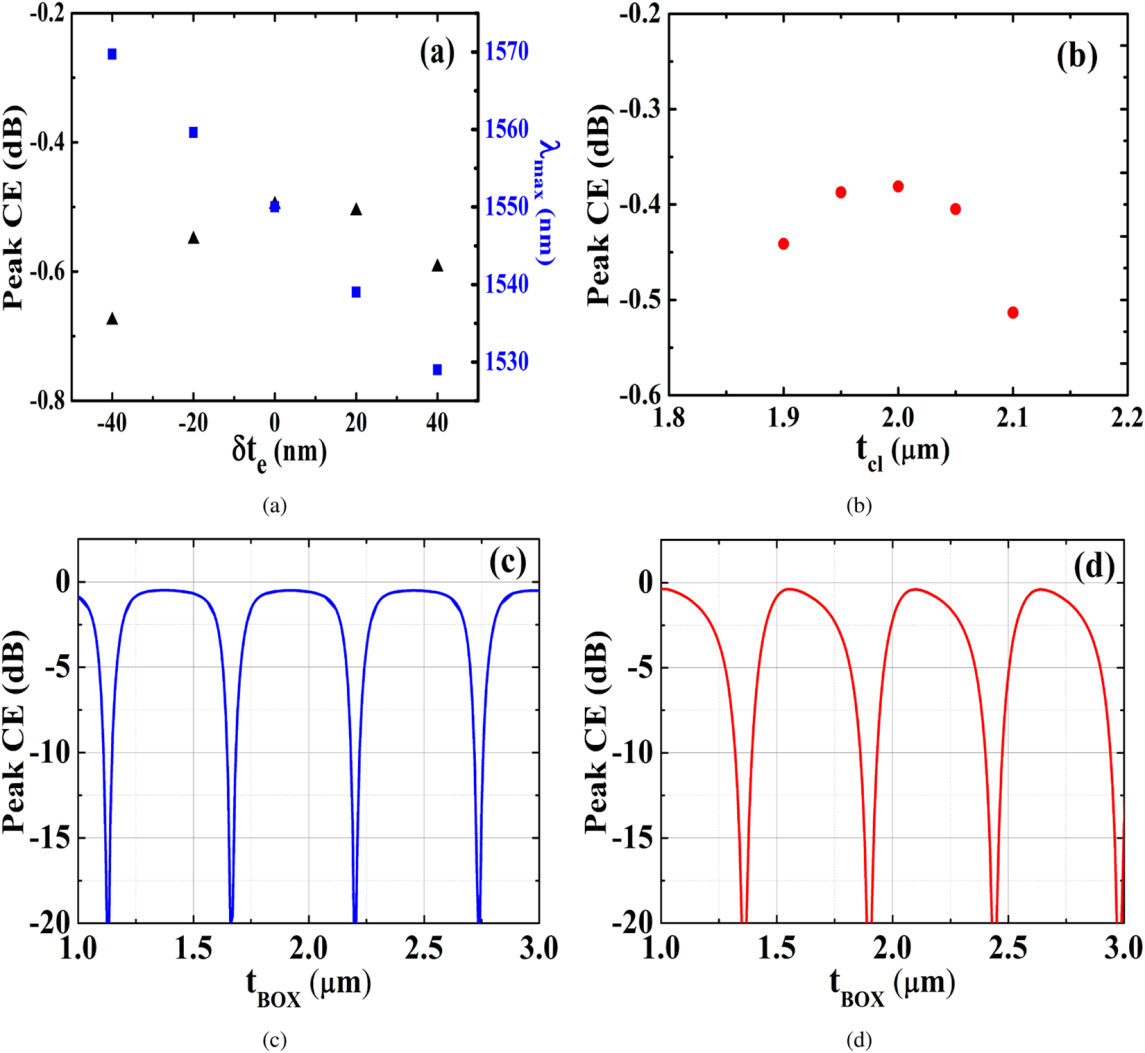
Coupling dependence on critical design parameters. (**a**) Shows dependence of etch depth variation *δt*_*e*_ on peak coupling and wavelength for design A and (**b**) shows dependence of upper cladding thickness *t*_*cl*_ on peak coupling for design B. (**c**) Shows the BOX thickness *t*_*BOX*_ impact on peak coupling performance for design A and (**d**) shows the same for design B.

From the fabrication perspective, a critical parameter is the quantity ${f}_{e}^{c}\Lambda$, which determines the minimum grating trench width. At optimum coupling, the minimum trench width for design A is 142 nm and for design B is 148 nm, both of which are feasible with current state of art 193 nm immersion deep UV lithography^32^. In any foundry, deviations from ideal design may inevitably occur and so it is necessary to estimate tolerance of fabricated structures to such process induced imperfections. Figure 3 outlines the impact of critical design parameters on the overall device coupling performance. At first, we take a look at two process related parameters, which are, etch depth for design A and upper cladding *SiO*_2_ thickness for design B. The etch depth variation and its effect on coupling and peak wavelength is plotted in Fig. 3(a). A peak wavelength shift of only 10 nm is observed for every 20 nm change in etch depth. In addition, we also note that for a ±40 nm etch depth variation, reduction in peak coupling is only about −0.17 dB. In case of the upper cladding thickness of design B (as shown in Fig. 3(b)), a ±100 nm deviation leads to a reduction of only −0.14 dB in peak coupling with no significant change in peak wavelength. Next, we take a look at the BOX thickness variation for both designs, which is plotted in Fig. 3(c,d). For design A, tolerance to *t*_*BOX*_ deviation is observed to be higher. For a ±100 nm variation, the change in peak coupling is only −0.15 dB. Compared to that, design B shows a lower tolerance to *t*_*BOX*_ variation. Here, for a ±50 nm deviation, the peak coupling is observed to change by −0.26 dB. These figures are however, within the standard limitations of photonic foundry processes and underline design robustness.

The coupling is also impacted by the number of DBR stacks. In Supplementary Fig. S1, this dependence is plotted. For design A, the difference in peak coupling between a *Si* substrate and a single layer DBR stack is only −0.81 dB. However for design B, this difference is significantly higher at −1.72 dB. Peak coupling between a single and double layer DBR stack differs by only −0.15 dB for design A, and by 0.35 dB for design B. For a 4 layer DBR substrate, calculated peak coupling is −0.45 dB for design A and −0.31 dB for design B. For either designs, the coupling appears to saturate beyond a 4 layer DBR.

A look at the scattered fields (as shown in Supplementary Fig. S2) shows an enhanced overlap of the fiber mode with the grating fields of both the CGA optimized designs, which would explain their high coupling performance.

## Alternative Combinations

As mentioned previously, the choice of *SiN* core thickness or top cladding is determined by its application. Keeping that in mind, we proceeded to implement the CGA on alternative *SiN* slabs of low and high thickness. Essentially two types of platforms are considered i.e., a partial etched air-clad grating and a fully etched *SiO*_2_-clad grating. The *SiN* waveguide slab thicknesses considered are for 300, 600 and 700 nm. In addition to these, we also looked at the converse of designs A and B i.e., 400 nm slab for partially etched gratings and 500 nm slab for fully etched gratings. The coupling performance of these designs are depicted in Supplementary Figs. S3 and S4 with a summary being provided in Table 2. A key observation is that, air-clad gratings with *Si* substrate of a given *SiN* slab thickness, have a substantially higher coupling on account of strong grating contrast. However, in the case of a 2 or 4 layer DBR stack substrate, the corresponding *SiO*_2_-clad, fully etched gratings, exhibit a comparatively higher coupling and bandwidth. For either platforms, a general trend to be noticed is the bandwidth reduction with increasing slab thickness, which can be attributed to the higher effective grating index of the fundamental mode^33^. The difference in peak CE between a 2 and 4 layer DBR is observed to be ~0.02–0.1 dB higher, for air-clad gratings and about ~0.07–0.1 dB higher, in the case of *SiO*_2_-clad gratings with negligible change in bandwidth. Maximum coupling among all combinations for a 2 layer DBR stack is observed for 500 nm thick *SiN* with *SiO*_2_-clad where a peak CE of −0.33 dB is calculated. On the other hand minimum peak coupling is calculated for an air-clad grating on 300 nm *SiN* slab, where peak CE is observed to be −0.65 dB.

**Table 2:** Peak CE and 1 dB bandwidth comparison of CGA optimized gratings for combinations of different *SiN* slab thickness and claddings.

*t*_*SiN*_ (nm)	Air cladding						*SiO*_2_ cladding					
	Peak Coupling (dB)			Bandwidth-*δλ*_1*dB*_ (nm)			Peak Coupling (dB)			Bandwidth-*δλ*_1*dB*_ (nm)		
	*Si* sub	2 DBR	4 DBR	*Si* sub	2 DBR	4 DBR	*Si* sub	2 DBR	4 DBR	*Si* sub	2 DBR	4 DBR
300	−3.4	−0.66	−0.56	27	35	36	−3.0	−0.56	−0.48	32	44	45
40	−2.2	−0.56	−0.5	33	39	40	−2.5	−0.38	−0.31	35	42	43
500	−1.47	−0.5	−0.46	31	33	33	−2.4	−0.33	−0.26	33	43	43
600	−1.2	−0.5	−0.48	24	26	26	−3.13	−0.36	−0.28	26	38	39
700	−1.65	−0.5	−0.46	22	23	23	−2.8	−0.46	−0.36	20	30	30

The figures are provided for 3 cases which are gratings with a bare *Si* substrate at optimal BOX height (*Si* sub), and those with a 2 and 4 layer DBR stack.

## Results and Discussion

**Figure 4:**
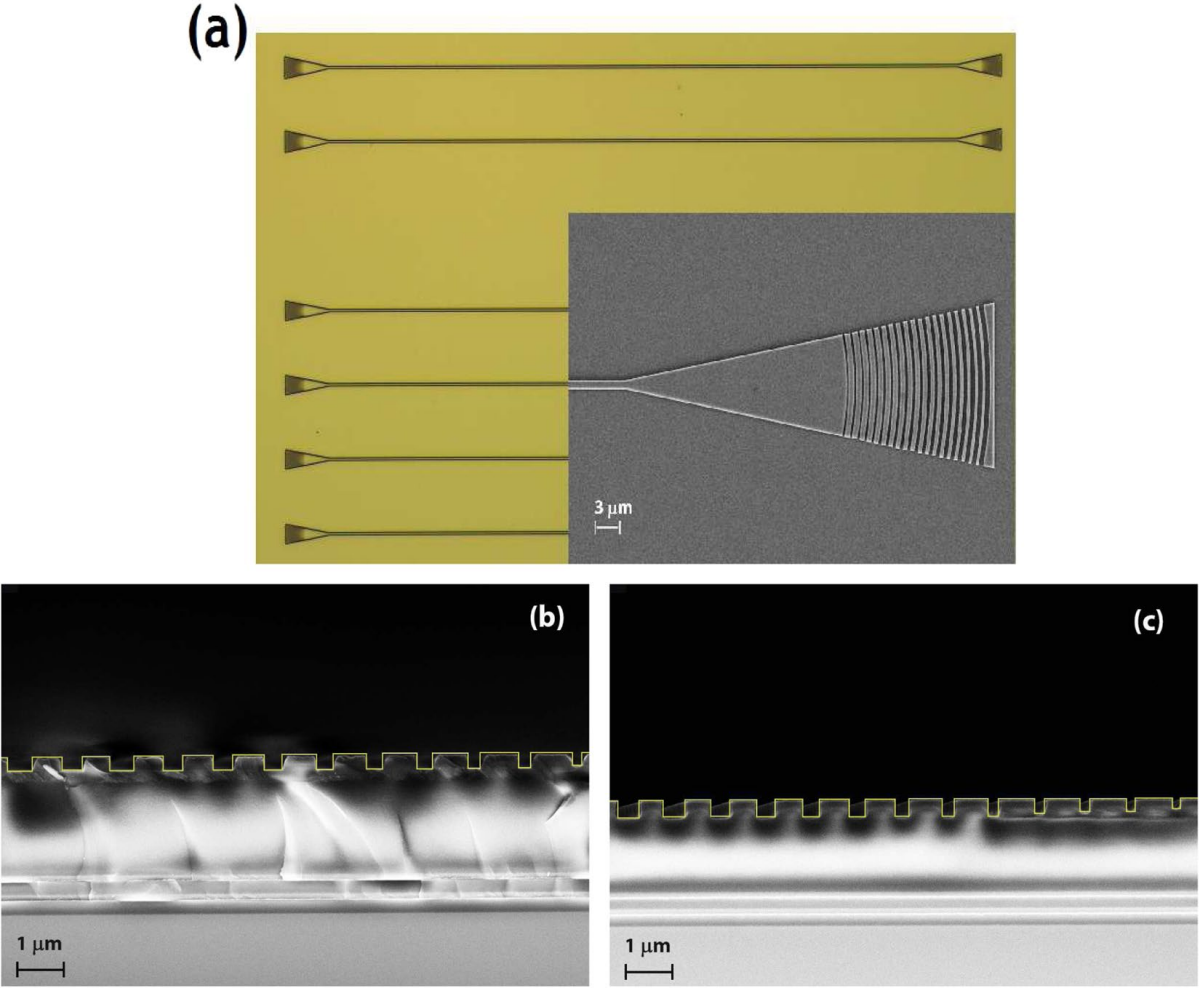
(**a**) Shows top view of some of the fabricated grating couplers. Inset shows an SEM image of one of the chirped focussing gratings. (**b** and **c**) Show side-view cross sectional SEM images of the etched grating couplers (see yellow trace) with the underneath DBR stack for (**b**) partial etched gratings of design A and (**c**) fully-etched gratings of design B prior to *SiO*_2_ cladding deposition.

**Figure 5:**
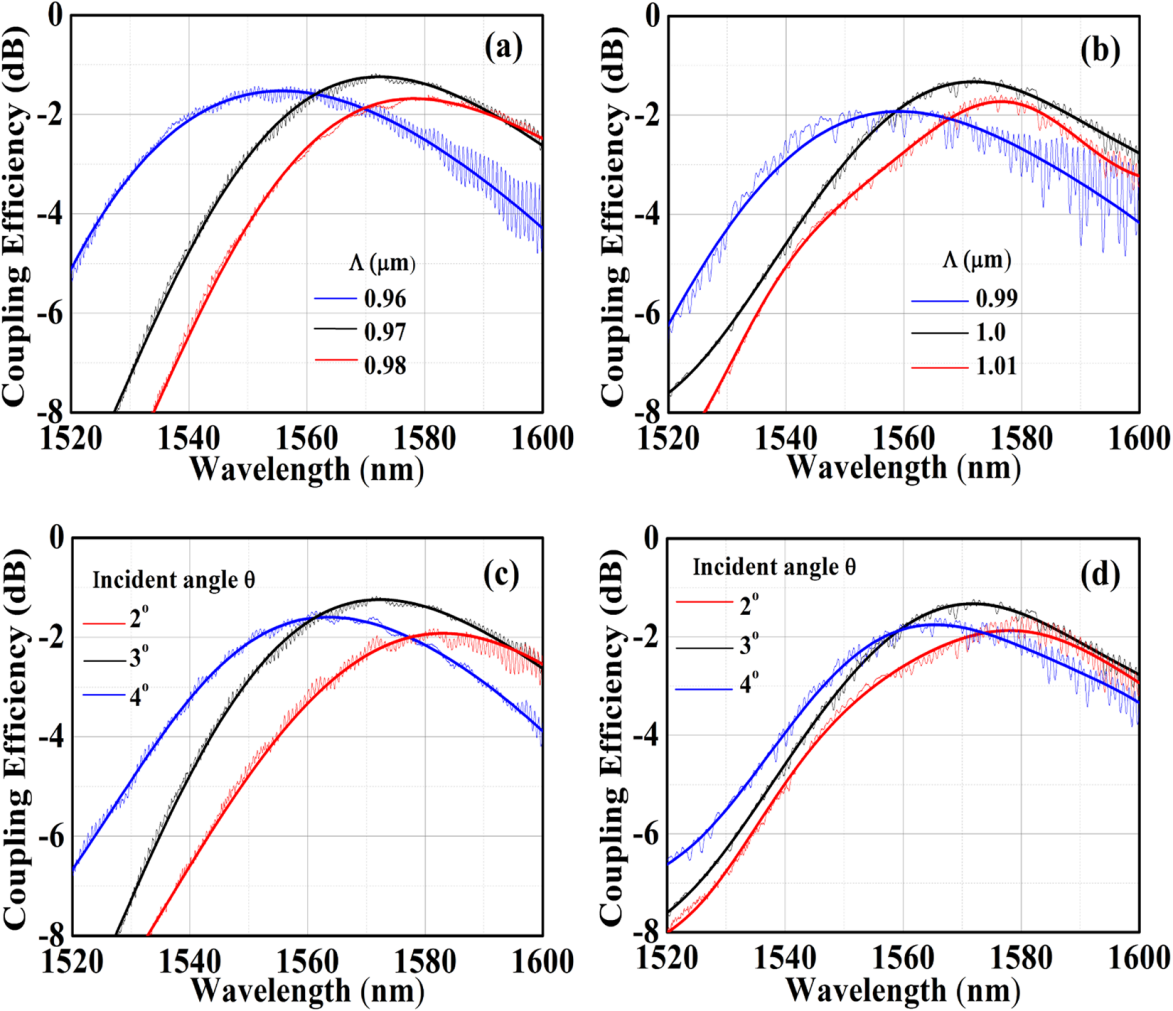
Experimental characterization of test devices. (**a**) Shows measured spectrum for design A at different periods and (**b**) shows the same for design B. (**c**) Depicts the angle dependence at peak period for design A and (**d**) shows the same for design B. The thin lines are the raw data and the thick lines represent a polynomial fit.

Figure 4 depicts the top view and side cross-sectional images of some of the fabricated test devices. The cross-sectional images reveal a slight under-etch for the front-end, low fill factor periods in both designs, which can be attributed to the lag effect. The results of characterization is plotted in Fig. 5. For design A, peak coupling is observed at −1.43 dB (1552 nm), −1.17 dB (1571 nm) and −1.61 dB (1580 nm) for periods of 0.960, 0.970 and 0.980 *μm* respectively. The corresponding 1 dB bandwidths are 45 nm, 40 nm and 43 nm. For design B, the peak coupling efficiencies are −1.83 dB (0.1561 nm), −1.24 dB (1572 nm) and −1.59 dB (1577 nm) at periods of 0.99, 1.0 and 1.01 *μm* with the 1 dB bandwidths being 46 nm, 39 nm and 33 nm respectively. For either designs, peak coupling is observed at 3° which also agrees with simulated data. The ripples in the measured spectrum are a consequence of reflections due to Fabry-Perot cavity effect, due to the short waveguide length. It may be mentioned that for the test devices, we chose a waveguide separation length of 700 *μm*. This was done to minimize contribution from propagation losses, while at the same time keeping a decent separation to avoid collision between the gonio-stage fibers at near vertical angles. The peak measured grating efficiency of the partially etched design A is observed to be slightly higher than those of design B. This is in contradiction to the simulated data which shows the fully etched gratings of design B, having a higher efficiency. Our analysis reveals that the under-etching of the front-end narrow trenches, which is more prominent in design B, to be the likely cause of this mismatch. The details of this analysis are provided in Supplementary Figs. S5 and S6. Table 3 outlines the current state of art for grating coupler demonstrations on different *SiN* platforms.

**Table 3:** Peak CE and 1 dB bandwidth comparison of different grating coupler designs demonstrated on various *SiN* platforms in S-C-L bands.

*SiN* Coupler	Max. CE (dB)	Bandwidth-*δλ*_1*dB*_ (nm)	*λ*_*max*_ (nm)	*t*_*SiN*_ (nm)	Clad	Etch step(s)	Bottom mirror	Platform
Ref. ^25^	−1.3	80	1536	400	*SiO*_2_	2	N	Non-Uniform, SiN/SOI
Ref. ^26^	−2.5	65	1564	600	air	1	N	
Ref. ^27^	−2.5	53	1484	400	*SiO*_2_	1	Y	Uniform, SiN
Ref. ^34^	−3.7	54	1555	700	air	1	N	
Ref. ^35^	−4.2	67	1570	400	*SiO*_2_	1	N	
Ref. ^29^	−2.29	49	1573	500	air	1	Y	
Ref. ^29^	−2.58	52	1576	400	*SiO*_2_	1	Y	
Ref. ^23^	−1.5	60 (3 dB)	1555	600	air	2	N	Non-Uniform, SiN
Ref. ^28^	−1.75	76.34 (3 dB)	1550	325	*SiO*_2_	1	N	
Ref. ^24^	−2.56	46.9	1550	220	*SiO*_2_	2	N	
**This work**	−**1.17**	**40**	**1571**	**500**	**air**	**1**	**Y**	
**This work**	−**1.24**	**39**	**1572**	**400**	**SiO**_**2**_	**1**	**Y**	

## Conclusion

In summary, we have demonstrated a novel scheme for grating couplers on *SiN* photonic chips. The scheme employs a chirp algorithm for gratings and a bottom Bragg reflector that can be implemented on a multitude of standalone *SiN* platforms. We have fabricated and characterized high efficiency chirped grating couplers on 2 distinct *SiN* platforms. Experimental peak efficiency for air-clad, partially etched grating on a 500 nm thick *SiN* is measured to be 1.17 dB/coupler at 1571 nm, with a 1 dB bandwidth of 40 nm. The corresponding efficiency for a fully etched grating on 400 nm thick *SiN*, with *SiO*_2_-cladding is measured to be 1.24 dB/coupler at 1572 nm, with a 1 dB bandwidth of 39 nm. The designs have minimum features that are compatible with scalable deep UV lithographic systems which have already been reported. Furthermore, we also demonstrate design robustness to significant process induced variations. Moreover, through simulations we also show that the chirping algorithm can deliver sub-dB coupler efficiency on a total of 10 different *SiN* chip combinations, comprising films between 300–700 nm thickness as well as surrounding claddings. These results pave way for implementing high performance gratings for a numerous applications concerning *SiN* platforms.

## Methods

### Simulation tools

All simulations were performed through a commercial FDTD solver from Lumerical Inc.

### Sample preparation and Characterization

Device fabrication is described as following. A DBR stack (specifications described in section 2) is deposited on a bare *Si* wafer, after which a piece each of the same sample is used for designs A and B. For design A, a BOX layer of 1.92 *μm* is deposited followed by an *SiN* slab layer of 500 nm, both of which are deposited using plasma-enhanced chemical vapour deposition (PECVD). The process is repeated for design B with corresponding optimized BOX and slab values. For gratings, we implement a focussing design with the optimized periods and CGA fill factors obtained from Table 1. The length of the waveguide is chosen to be 700 *μm*. All patterns are written using electron beam lithography (Raith eline) with a negative resist (MaN 2403). The dimensions of rib waveguide for design A are 1.2 × 0.28 *μm* and of ridge waveguide for design B are 1.1 × 0.4 *μm*. The patterns are subsequently etched using inductively coupled reactive-ion etching (ICP-RIE, Oxford Systems) using fluorine etch chemistry. For design B, subsequent to the etching process, a top cladding layer of *SiO*_2_ of 2 *μm* thickness is deposited using PECVD. Further information on the fabrication process is available in^29^. The fabricated devices are characterized using a tunable laser source (Keysight 8146 B). SMF fibers connect the sample mounted gonio stage via polarization controllers to the laser source. The propagation loss measured through cutback method is found to be 0.5 dB/mm for design A and 0.4 dB/mm for design B. These figures are deducted from the final fiber to fiber transmission.

## Supplementary information

Supplementary Information
