# Supplementary material for: High efficiency DBR assisted grating chirp generators for silicon nitride fiber-chip coupling
Source: Sci Rep. 2019 Dec 11;9:18821. doi: 10.1038/s41598-019-55140-8 (PMC6906413; doi:10.1038/s41598-019-55140-8)
Supplement: Supplementary file 1 — Supplementary Information [file 41598_2019_55140_MOESM1_ESM.pdf]

Supplementary Information for

High Efficiency DBR assisted Grating Chirp Generators for Silicon Nitride Fiber-Chip coupling

Siddharth Nambiar[1,*], Praveen Ranganath[1], Rakshitha Kallega[1] & Shankar Kumar Selvaraja[1]

[1] *Center for Nanoscience and Engineering, Indian Institute of Science, Bengaluru, 560012, India*

[*] *sidnam12@gmail.com*

Impact of DBR stacks

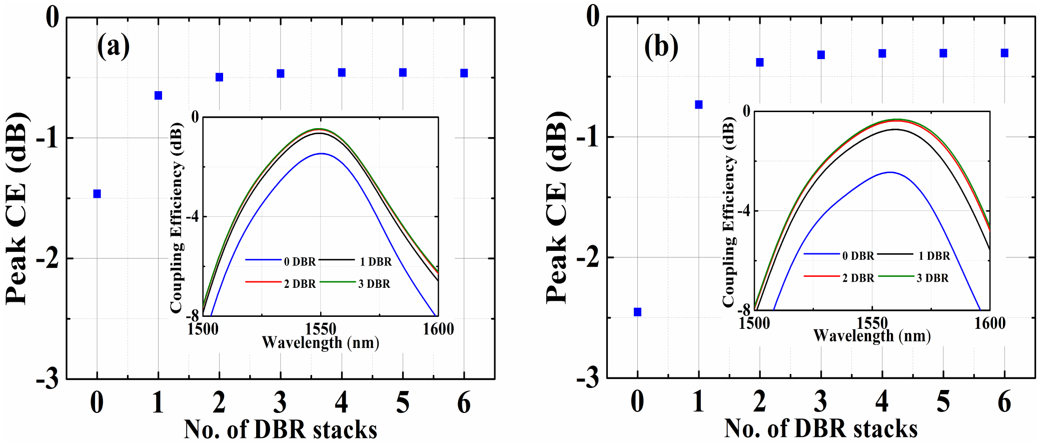

Figure S 1: Dependence of peak CE on the number of DBR stacks for (a) design A and (b) design B. 0 DBR corresponds to a bare Si substrate. Inset shows the coupling spectrum for different DBR stacks.

Field plot

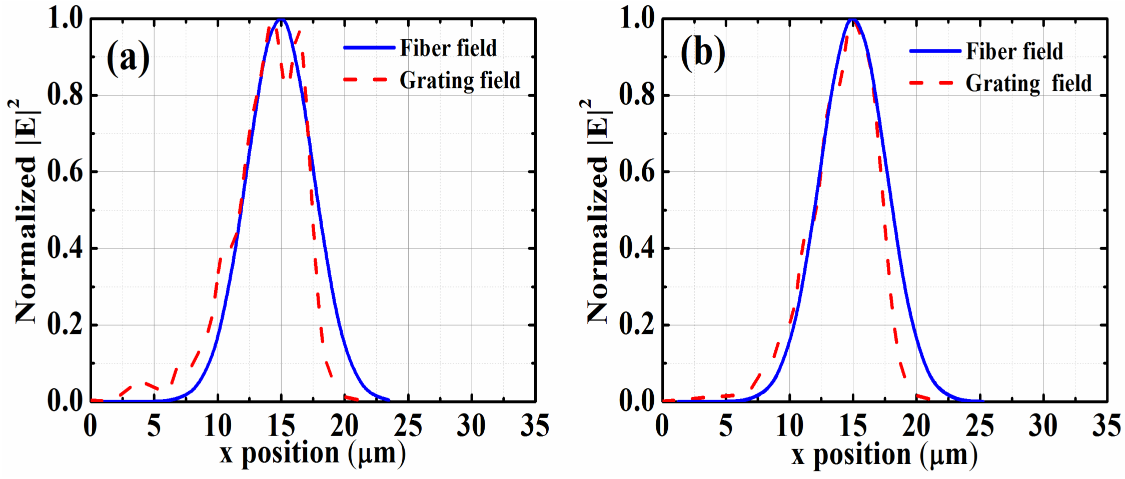

Figure S 2: (a) The normalized scattered grating fields for CGA optimized, partially etched gratings of design A and (b), fully etched gratings of design B. For comparison, the fiber mode field profile is also plotted.

Coupling performances of alternative chirped combinations

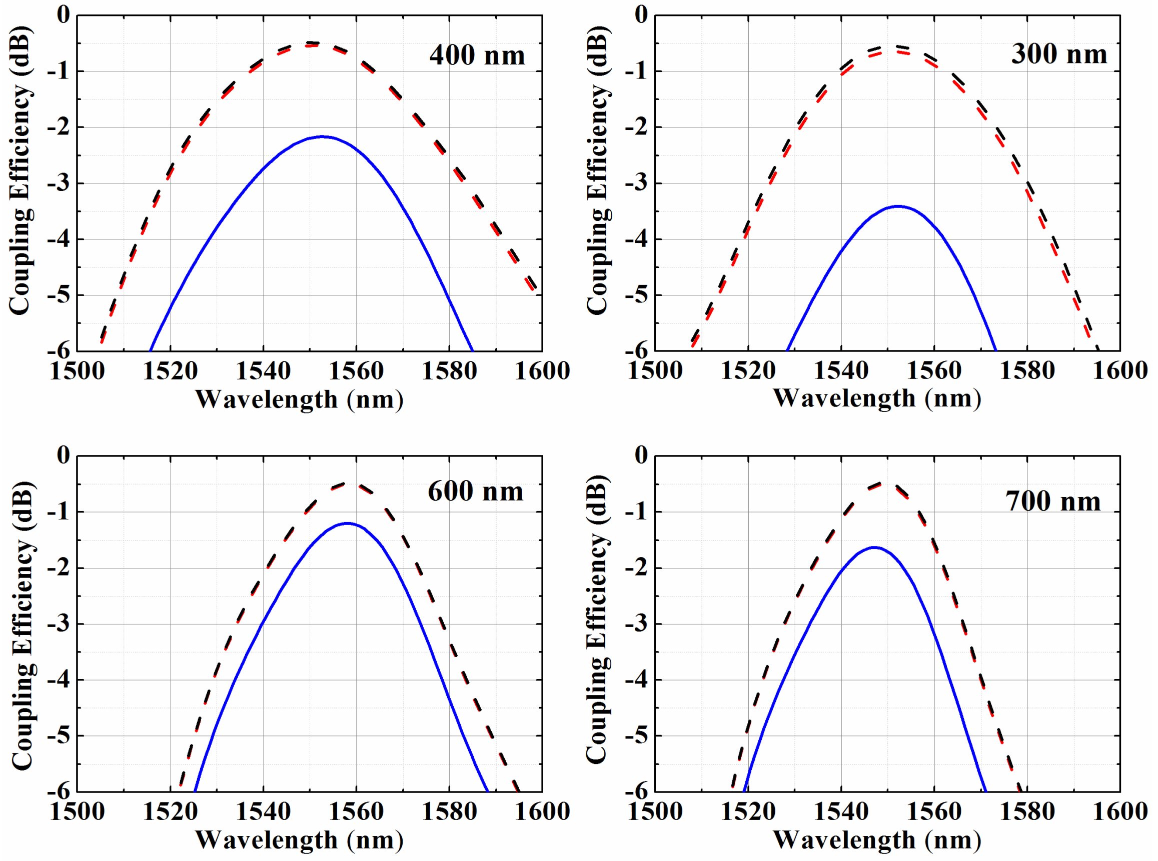

Figure S 3: Coupling performances of all air-clad, partially etch grating designs of different SiN slab thickness. The solid blue line legend is for a grating on a bare Si substrate and the red and black dashed lines are for 2 and 4 layered DBR stacks respectively.

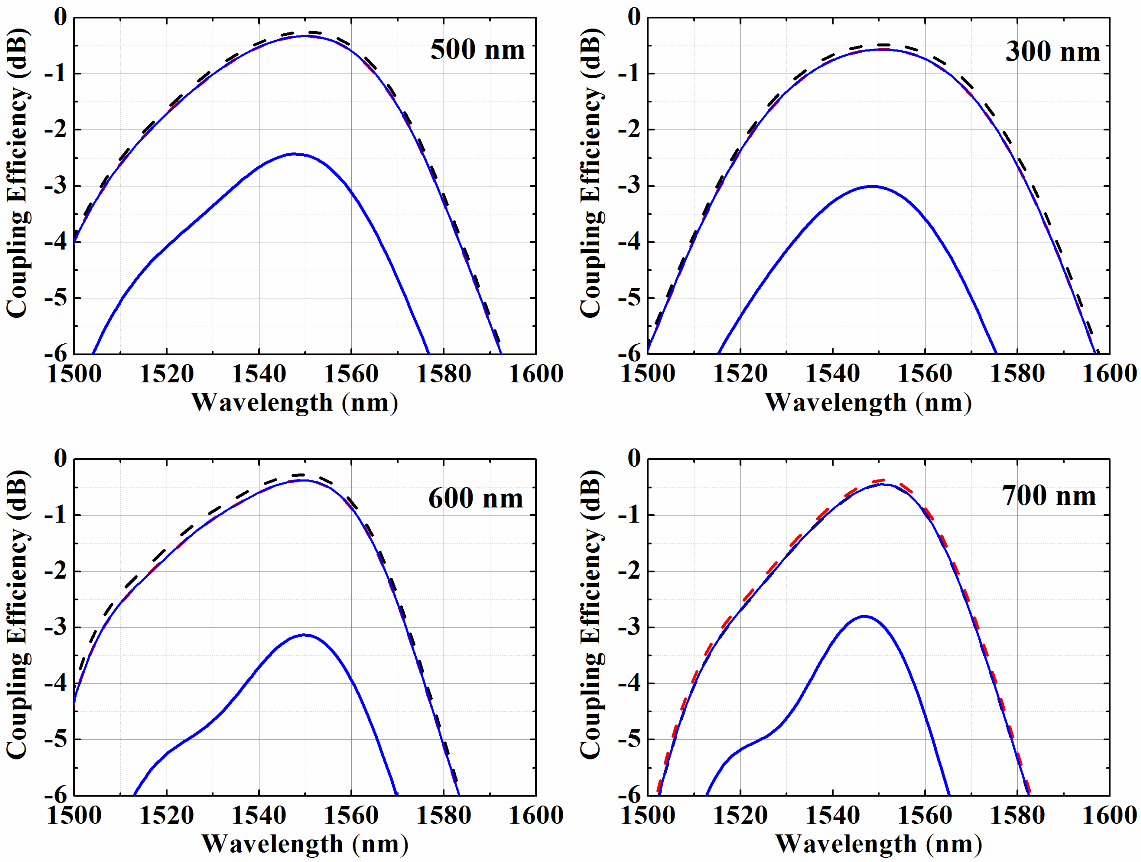

Figure S 4: Coupling performances of all SiO_2-clad fully etched grating designs. The plot legends being the same as described above in Fig. S3.

Effect of etch lag on coupling performance

The etch lag effect was analysed by implementing an etch ramp down function on the CGA optimized designs. The ramp down is depicted in figure S5.

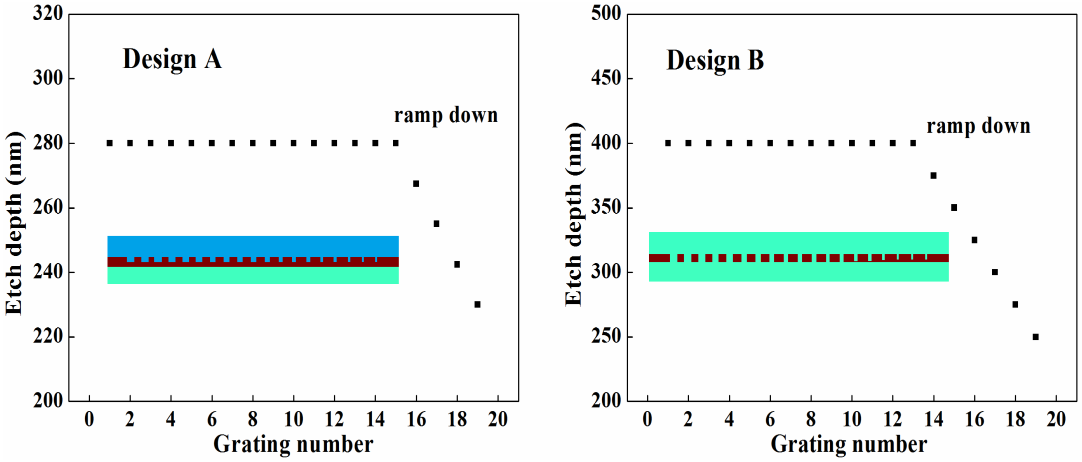

Figure S 5: Etch depth variation applied to CGA optimized gratings. The ramp down function was applied to a 50 nm under-etch over the front-end 5 periods for design A and a 150 nm under-etch over the front-end 7 periods for design B. These estimates are based on cross-sectional SEM images. Inset in each plot shows the optical index profile of the simulated gratings.

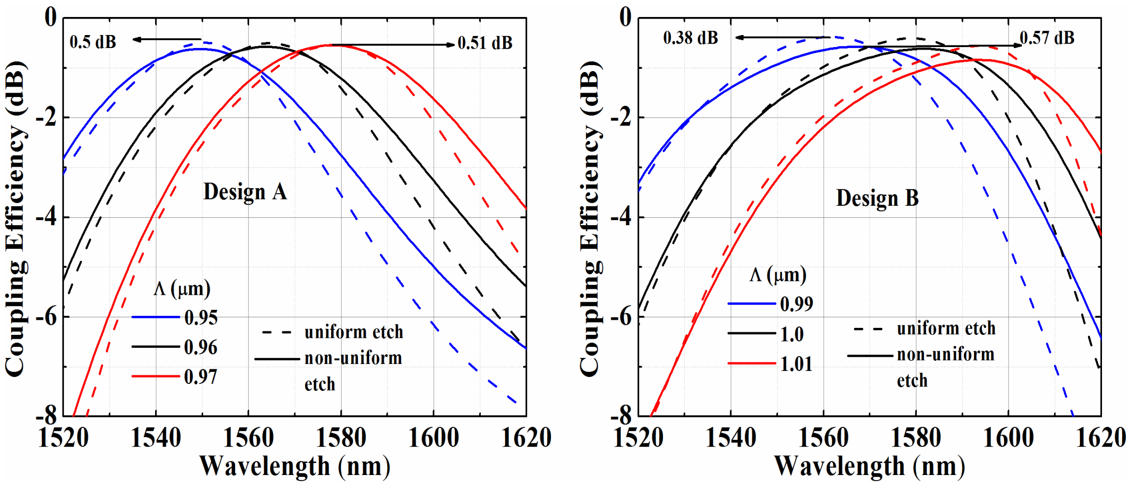

Figure S 6: Simulated coupling performance comparison of CGA optimized uniform etch gratings and those of non-uniform etch profile with the ramp down function, at different periods.
